# Resilience and mindfulness among radiological personnel in Norway, their relationship and their impact on quality and safety– a questionnaire study

**DOI:** 10.1186/s13104-024-06748-1

**Published:** 2024-04-01

**Authors:** Ann Mari Gransjøen

**Affiliations:** 1 Department of Health Sciences in Gjøvik, Norwegian University of Science and Technology in Gjøvik (NTNU), Teknologiveien 22, 2815 Gjøvik, Norway; 2 SHARE-Centre for Resilience in Healthcare, Faculty of Health Sciences, University of Stavanger, Kjell Arholmsgate 41, 4036 Stavanger, Norway

**Keywords:** Individual resilience, Organizational resilience, Mindfulness, Quality and safety

## Abstract

**Background:**

Stress and burnout are widespread problems among radiological personnel Individual and organizational resilience and mindfulness offer protection against burnout.

**Aim:**

To investigate the level of resilience and mindfulness among radiological personnel, the associations between organizational resilience, individual resilience, and mindfulness, and how these factors impact the quality of care provided in radiological departments.

**Methods:**

An online questionnaire consisting of the Connor-Davidson Resilience Scale, the Mindful Attention Awareness Scale, the Benchmark Resilience Tool, and questions regarding burnout, and quality and safety was used. Data analysis consisted of descriptive statistics, bivariate correlation and standard multiple regression.

**Results and Conclusion:**

Few participants considered burnout a significant challenge. Individual and organizational resilience were low (30.40 ± 4.92 and 63.21 ± 13.63 respectively), and mindfulness was high (4.29 ± 0.88). There was a significant correlation between individual and organizational resilience (*p* = 0.004), between individual resilience and mindfulness (*p* = 0.03), and between organizational resilience and mindfulness (*p* = 0.02). Individual and organizational resilience affect each other. However; neither significantly affect quality and safety, nor mindfulness

**Supplementary Information:**

The online version contains supplementary material available at 10.1186/s13104-024-06748-1.

## Introduction

Stress and burnout are widespread problems among radiological personnel [1–11]. Different forms of mindfulness, ranging from formal meditation to more informal attention to day-to-day tasks, can prevent and reduce burnout among radiological personnel [3]. Another strategy for reducing stress and burnout among health professionals is promoting individual resilience [4, 9, 12]. Mindfulness and resilience could also affect the quality of care, due to their effectiveness in reducing stress and burnout [2, 5, 13].

Organizational resilience regards an organization’s ability to manage change, bounce back from setbacks and maintain desirable functions and outcomes under pressure. This is influenced by for example leadership practices and human capital [14]. Some studies show a link between individual resilience and organizational resilience, and that these two types of resilience affect each other [15–17].

The objective of this study is to investigate the level of resilience and mindfulness among radiological personnel, the associations between organizational resilience, individual resilience, and mindfulness, and how these factors impact the quality of care provided in radiological departments.

## Main text

### Materials and methods

#### Design and setting

This study utilized a cross-sectional design to collect data on resilience, mindfulness, and the quality and safety of care among healthcare workers and their departments. The study is set within radiological departments in Norway, which encompasses both public hospitals and private institutions.

#### Population, study size and recruitment

The study population consisted of radiologists, registrars, radiographers, and radiation therapists. Participants were selected based on the following eligibility criteria; (a) they had a valid authorizations and (b) they currently worked in a clinical setting. According to an online sample size calculator (surveymonkey.com) the estimated sample size needed for this study, based on population size, 95% CI and 5% margin of error, was approximately 356 participants, which was not reached.

Participants were recruited in collaboration with the Norwegian Society of Radiographers and the Norwegian Radiological Association. These associations posted the link to a digital, online questionnaire on social media and their newsletter, resulting in probability sampling. Recruitment lasted from July 18th to October 5th 2022 and included a total of 4783 members.

#### Variables, data sources and measurement

The variables of interest in this study were individual resilience, mindfulness, organizational resilience, and quality and safety. Background variables that were used were public vs. private setting, and how leaders address burnout. All these variables were measured through a questionnaire consisting of six parts.

Not all parts had a Norwegian version available. The researcher, following the steps described by the Norwegian Directory of Health, translated this from English to Norwegian. Too see the interview guide used in the validation of the translated questionnaire, see supplementary file 2.

Part 1 was designed by the researcher to collect demographic data about the respondent. This included profession, workplace (public vs. private), department size and whether their position included personnel management.

Part 2 is the Norwegian Connor-Davidson Resilience Scale (CD-RISC-10), which is used to assess the ability to respond and adapt to life adversity, trauma, tragedy, threats or other major life stressors [18].

Part 3 is the five item Mindfulness Attention Awareness Scale (MAAS) translated to Norwegian by Smith et al. This scale measures the extent to which an individual can attend to, and remain aware of, experiences in the present moment [19]..

Part 4 is the short version of the Benhmark Resilience Tool (BRT 13), which assesses behavioral traits and perceptions linked to the organization’s ability to plan for, respond to, and recover from emergencies and crises (organizational resilience) [20].

Parts 5 and 6 are aimed at specific groups. Part 5 was intended for respondents with personnel management roles and was only made available for the respondents who answered they had such roles. These questions were inspired by the questionnaire developed by Parikh et al. (2020) to evaluate a leader’s effectiveness in detecting burnout among employees, and the tools used to measure burnout among employees [21].

Part 6 was intended for radiographers and radiotherapists and only made available for those listing these as their profession. The researcher designed the questions to evaluate the aspects of quality and safety in radiology that may be affected by stress and mindfulness. To see the questionnaire in its entirety, see supplementary file 1.

### Statistical analysis

All analyses were performed using IBM SPSS version 26.0. Cronbach’s α was measured to further validate the translated parts of the questionnaire. A low value could indicate poor translation.

**Fig. 1:**
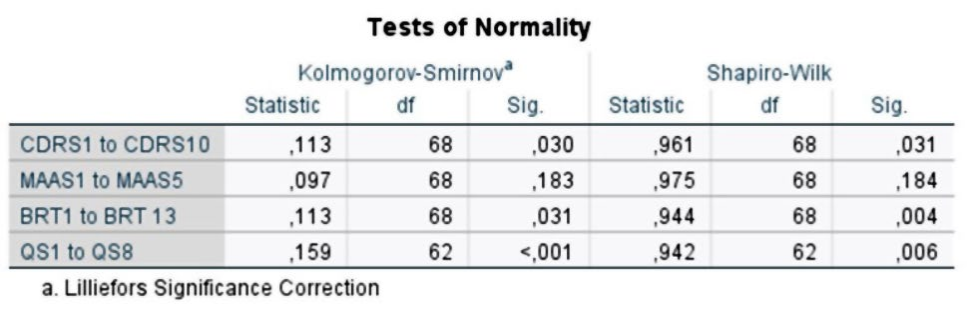
Tests of normality. Table produced by SPSS describing the tests of normality that were performed on all main variables: individual resilience (CDRS1 to CDRS10), mindfulness (MAAS1– MAAS5), organizational resilience (BRT1 to BRT13), and quality and safety (QS1 to QS8). This includes the Kolmogorov - Smirnov and Shapiro - Wilk tests. The significance value (Sig.) under 0.05 indicates that the variables individual resilience, organizational resilience and quality and safety are not normally distributed. This does not necessarily indicate a problem with the scale used, but rather reflects the underlying nature of the construct being measured. In the case of resilience previous studies have shown this to be low among radiological personnel, which can explain why this variable is somewhat skewed. Low organizational resilience can explain why this variable is skewed, and high quality and safety can explain why this variable is skewed even if there are no problems with the scales themselves. Further inspections of normality are shown in figures 2, 3, 4 and 5

**Fig. 2:**
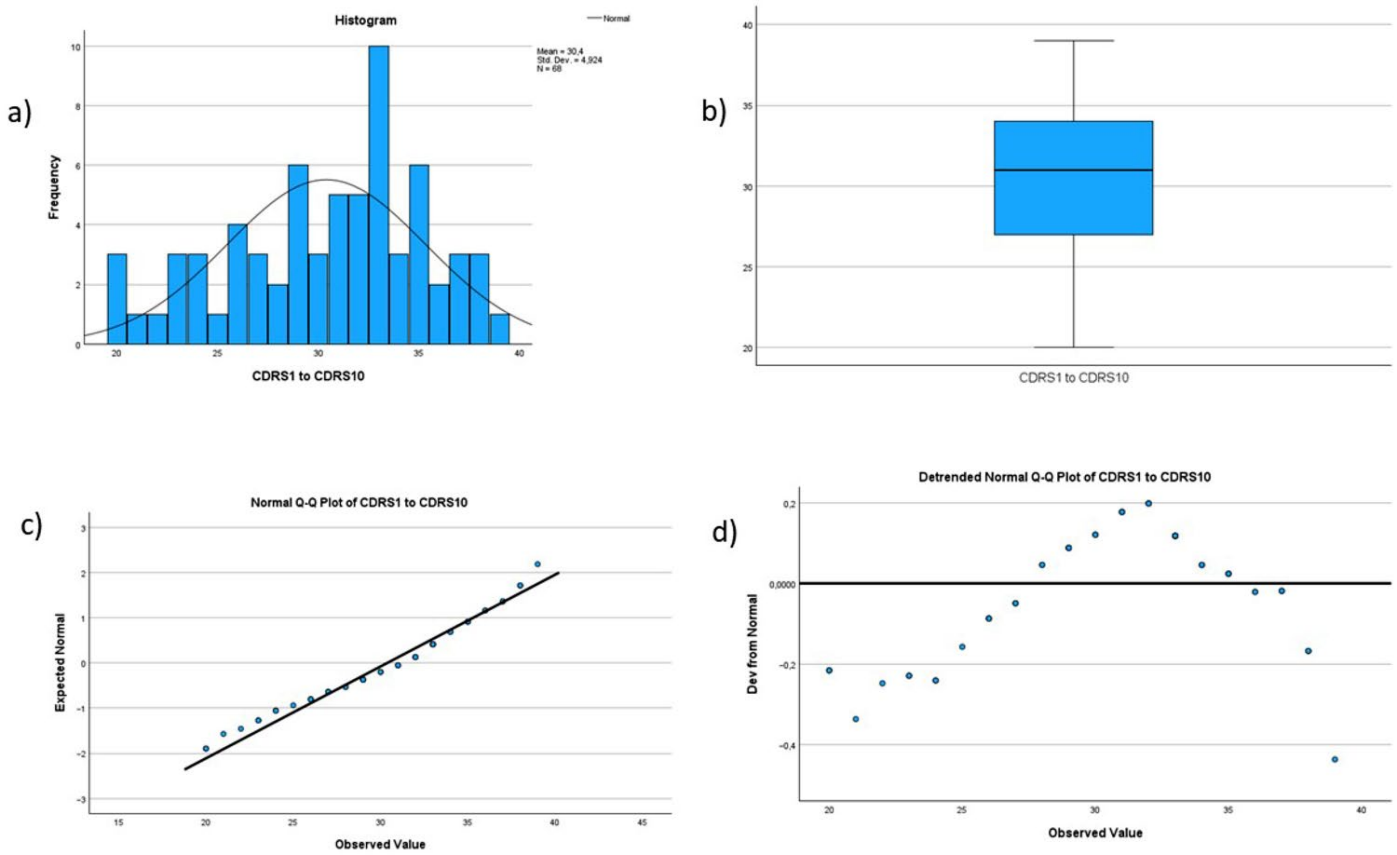
Histogram, boxplot, and Q-Q Plots for the variable individual resilience. The histogram (labeled a in the figure) shows that the data are not entirely normally distributed but have a peak to the left. However, the data are not severely skewed. The boxplot (labeled b in the figure) shows no outliers. The Normal Q-Q Plot (labeled c in the figure) shows a reasonably straight line, indicating that the data are not entirely normally distributed, but are not severely skewed. Last, the Detrended Normal Q-Q Plot (labeled d in the figure) show no clustering of points, indicating that the data are not severely skewed for this variable

**Fig. 3:**
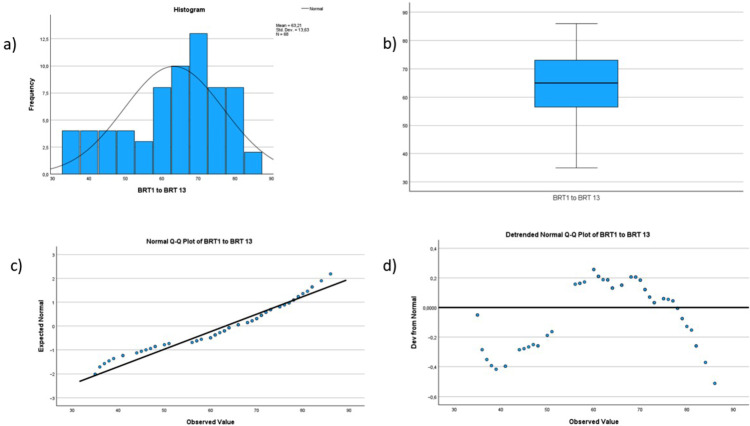
Histogram, boxplot and Q-Q Plots for the variable organizational resilience. The histogram (labeled a in the figure) shows that the data are not entirely normally distributed but are somewhat skewed to the left. However, the data are not severely skewed. The boxplot (labeled b in the figure) shows no outliers. The Normal Q-Q Plot (labeled c in the figure) shows a reasonably straight line, indicating that the data are not entirely normally distributed, but are not severely skewed. Last, the Detrended Normal Q-Q Plot (labeled d in the figure) show no clustering of points, indicating that the data are not severely skewed for this variable

**Fig. 4:**
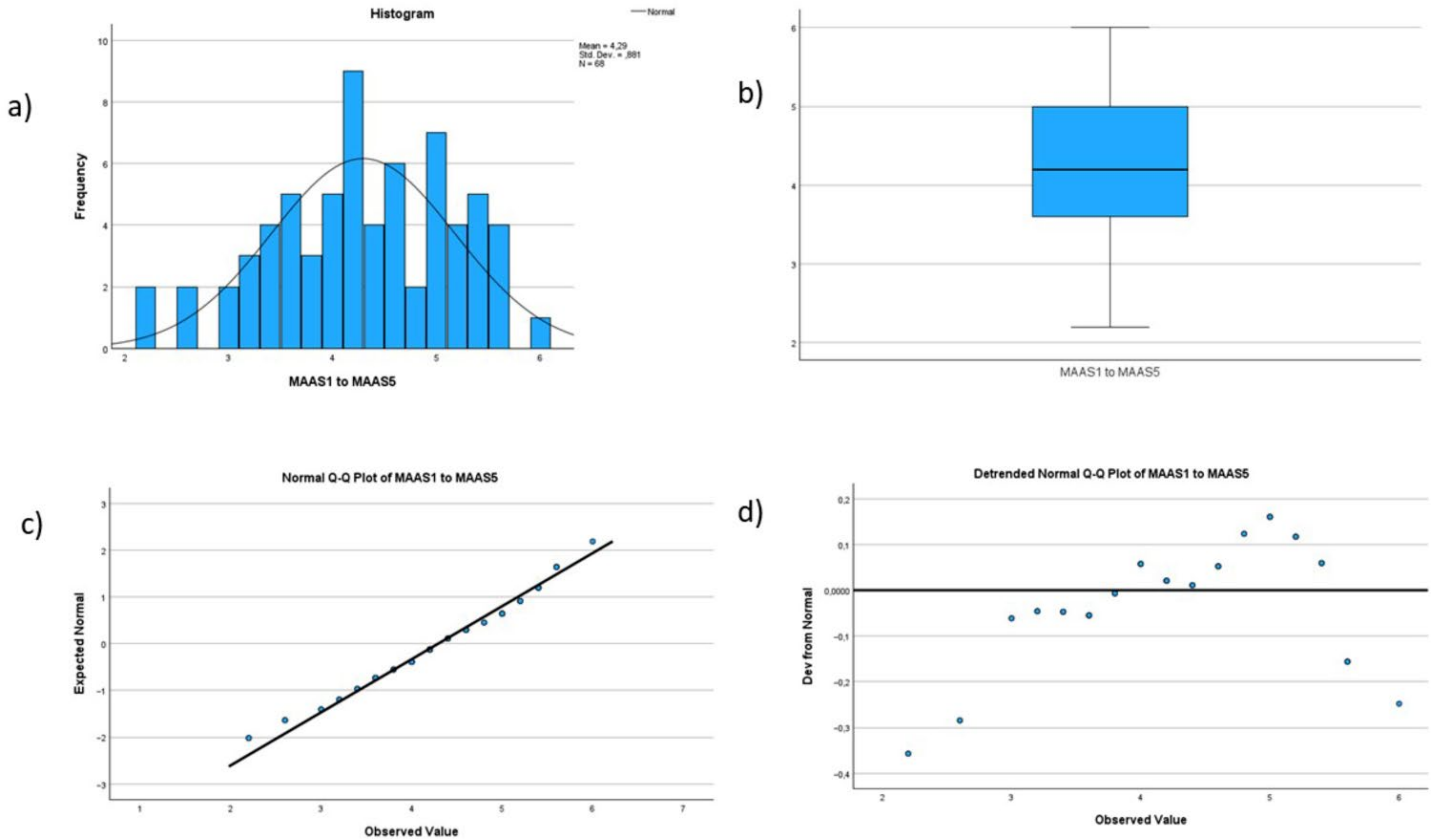
Histogram, boxplot and Q-Q Plots for the variable mindfulness. The histogram (labeled a in the figure) shows that the data are reasonably normally distributed. The boxplot (labeled b in the figure) shows no outliers. The Normal Q-Q Plot (labeled c in the figure) is showing a reasonably straight line, indicating that the data is normally distributed. Last, the Detrended Normal Q-Q Plot (labeled d in the figure) shows no clustering of points, indicating that the data are not skewed for this variable.

**Fig. 5:**
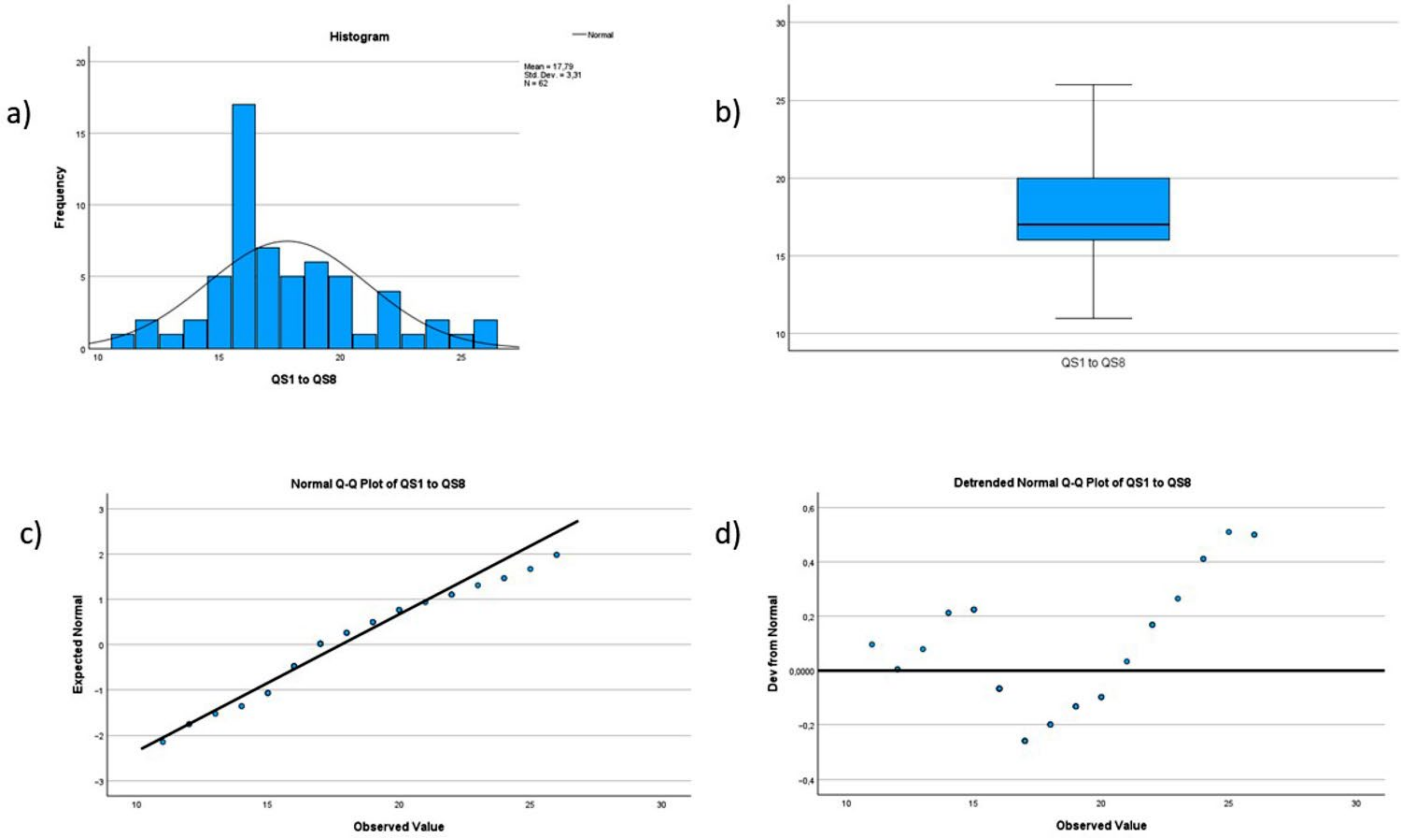
Histogram, boxplot and Q-Q Plots for the variable quality and safety. The histogram (labeled a in the figure) shows that the data are not entirely normally distributed but have a peak to the right. However, the data are not severely skewed. The boxplot (labeled b in the figure) shows no outliers. The Normal Q-Q Plot (labeled c in the figure) shows a reasonably straight line, indicating that the data are not entirely normally distributed, but are not severely skewed. Last, the Detrended Normal Q-Q Plot (labeled d in the figure) show no clustering of points, indicating that the data are not severely skewed for this variable.

Demographic data, the score for individual and organizational resilience and mindfulness are described using frequencies and means. See Figs. 1, 2, 3, 4 and 5 for tests of normality performed for all main variables. Bivariate correlation using Spearman’s rho was used for correlation analysis, and standard multiple regression was used to further explore the relationships between the variables.

Three models for multiple linear regression were used. In the first model, individual resilience was used as the dependent variable (is individual resilience affected by organizational resilience and mindfulness?). In the second model, organizational resilience was used (is organizational resilience affected by individual resilience and mindfulness), and in the third, quality and safety were used as the dependent variable (is quality and safety affected by both types of resilience and mindfulness?)

**Fig. 6:**
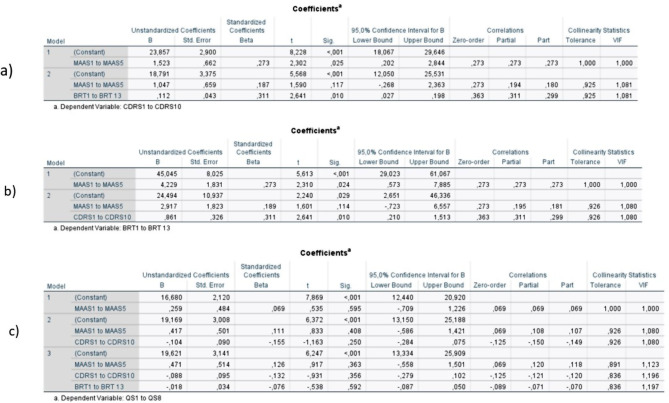
Tests for confounding factors in the models. To check for confounding factors the models were built by adding in one independent variable at a time. In model 1 (labeled a in the figure), where individual resilience is the dependent variable and mindfulness and organizational resilience are the independent variables, mindfulness might be a confounding variable. This is indicated by a change in the β-value (and standardized β-value) that is rather large. However, the large CI makes this change less worrisome. In model 2 (labeled b in the figure), where organizational resilience is the dependent variable and individual resilience and mindfulness are the independent variables a similar challenge occurred. This can indicate that the confounding might be between mindfulness and individual resilience. However, the CI is still large enough that the change in value in mindfulness is not worrisome. In the third and last model (labeled c in the figure), mindfulness still might be a confounding variable with individual resilience based on the change in its β-value when individual resilience is introduced which is not seen when organizational resilience is introduced to the model. The change in beta-value is the largest in this model, and the smaller CI makes this change more worrisome than in the other two models. The change in β-values and large CI can also, in part, be explained by the correlation between these factors and the relationship between them that has been established in previous studies. Since the evidence for confounding is not that strong and the indication of confounding is between two factors with a known correlation the choice was made to perform the statistical analysis as planned.

**Fig. 7:**
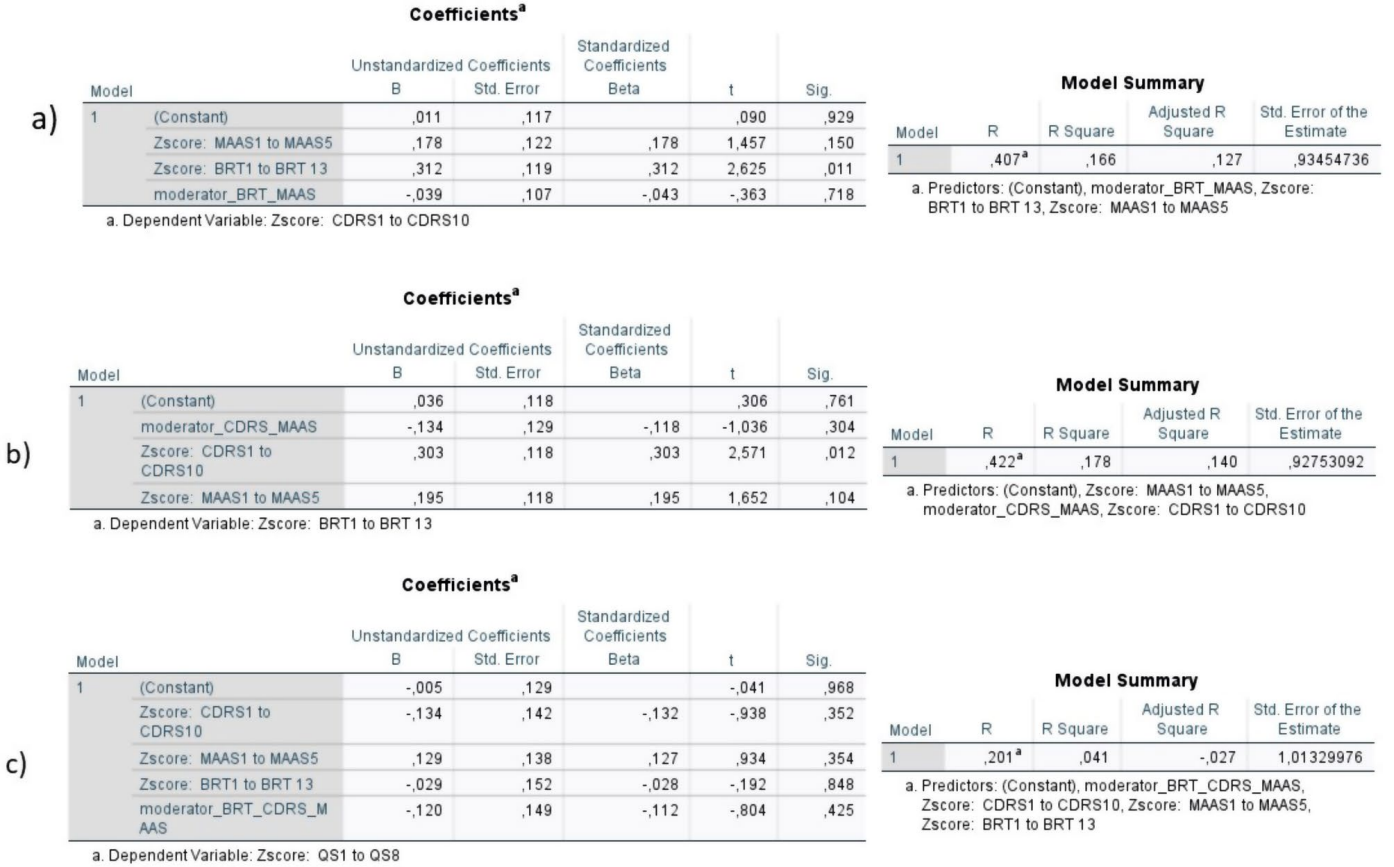
Tests for interacting variables. To check for interaction between variables the Z-scores for the variables were used, as well as moderator-variables. The Z-scores are a variable standardized to have a standard deviation of 1 and a mean of 0. The moderation-variable is the product of the independent variables in the planned regression model, which is then added to the regression model. To confirm if a variable has a moderation effect on the relationship between an independent variable and a dependent variable, the nature of this relationship must change once the moderator variable changes. In this case there does not seem to be any interacting factors, since the moderator variable is not statistically significant in either model 1 (labeled a in the figure), model 2 (labeled b in the figure) or model 3 (labeled c in the figure). This is further supported by the fact that the R Squared or adjusted R squared did not significantly change between this model and the model run with the actual variables, indicating that the relationship between the variables has not changed

**Fig. 8:**
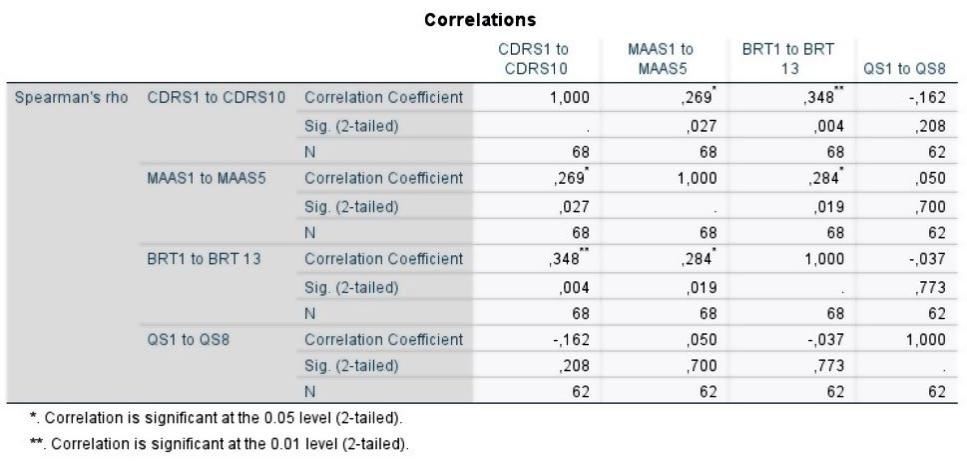
Bivariate correlation using Spearman’s Rho. The correlation analysis revealed that there are statistically significant relationships between mindfulness and individual resilience (ρ = 0.27, n=62, p= 0.03), between mindfulness and organizational resilience (ρ = 0.28, n=62, p= 0.02), and between individual and organizational resilience (ρ = 0.35, n=62, p= 0.004). There are no variables that are significantly correlated with quality and safety, however. Even if it is not statistically significant, there seems to be a small, negative relationship between quality and safety and individual resilience (ρ = -0.16, n=62, p=0.21). This could indicate that there is a relationship between these variables that could be worth exploring even if their relationship is not statistically significant in this test.

The model building supports the use of these models. Even if mindfulness might be a confounding factor with individual resilience (see Fig. 6 and limitations for the discussion of its effect), there are no obvious interacting variables (see Fig. 7), and bivariate correlation shows some relationship between most of the variables (see Fig. 8), in addition to the literature indicating that these variables have some effect on each other.

The significance level was set at *P* < 0.05 for all tests performed.

## Results

The Cronbach’s α scores ranged from 0.72 to 0.89, indicating internal consistency in all parts of the questionnaire. Thirty-one radiologists, 8 registrars, 24 radiographers and 5 radiotherapists completed the questionnaire (total = 68). Most respondents worked in a public setting (88%), and 67% worked in moderate to large departments. Eleven respondents (16%) had a personnel management role. Of those 11 respondents, 12.9% considered burnout a significant challenge among their employees. Approximately 1.7% of the respondents considered themselves to be very effective at detecting burnout, and 81% reported using a tool to detect employee burnout. The tools used were personal development interviews (55%), questionnaires (33%) and work environment surveys (11%).

The CD-RISC-10 total score was 30.40 ± 4.92, BRT 13 was 63.21 ± 13.63, and MAAS was 4.29 ± 0.88. The highest scores were for those working in the private sector. The total score for quality and safety was 17.79 ± 3.31. The public sector scored slightly lower than the private sector (17.83 vs. 18.20), and departments with the fewest labs (> 5) had the lowest score (16.00 ± 0.44), indicating higher quality.

The relationship between individual resilience, organizational resilience, mindfulness and quality and safety was investigated using bivariate correlation (Spearman’s rho is reported). This was chosen when preliminary analysis indicated some violations of normality (see Figs. 1, 2, 3, 4 and 5). There was a small, positive correlation between mindfulness and individual resilience (ρ = 0.27, *n* = 62, *p* = 0.03), and between mindfulness and organizational resilience (ρ = 0.28, *n* = 62, *p* = 0.02). There was also a moderate, positive correlation between individual and organizational resilience (ρ = 0.35, *n* = 62, *p* = 0.004). See Fig. 8 for more information obtained from the bivariate correlation.

**Fig. 9:**
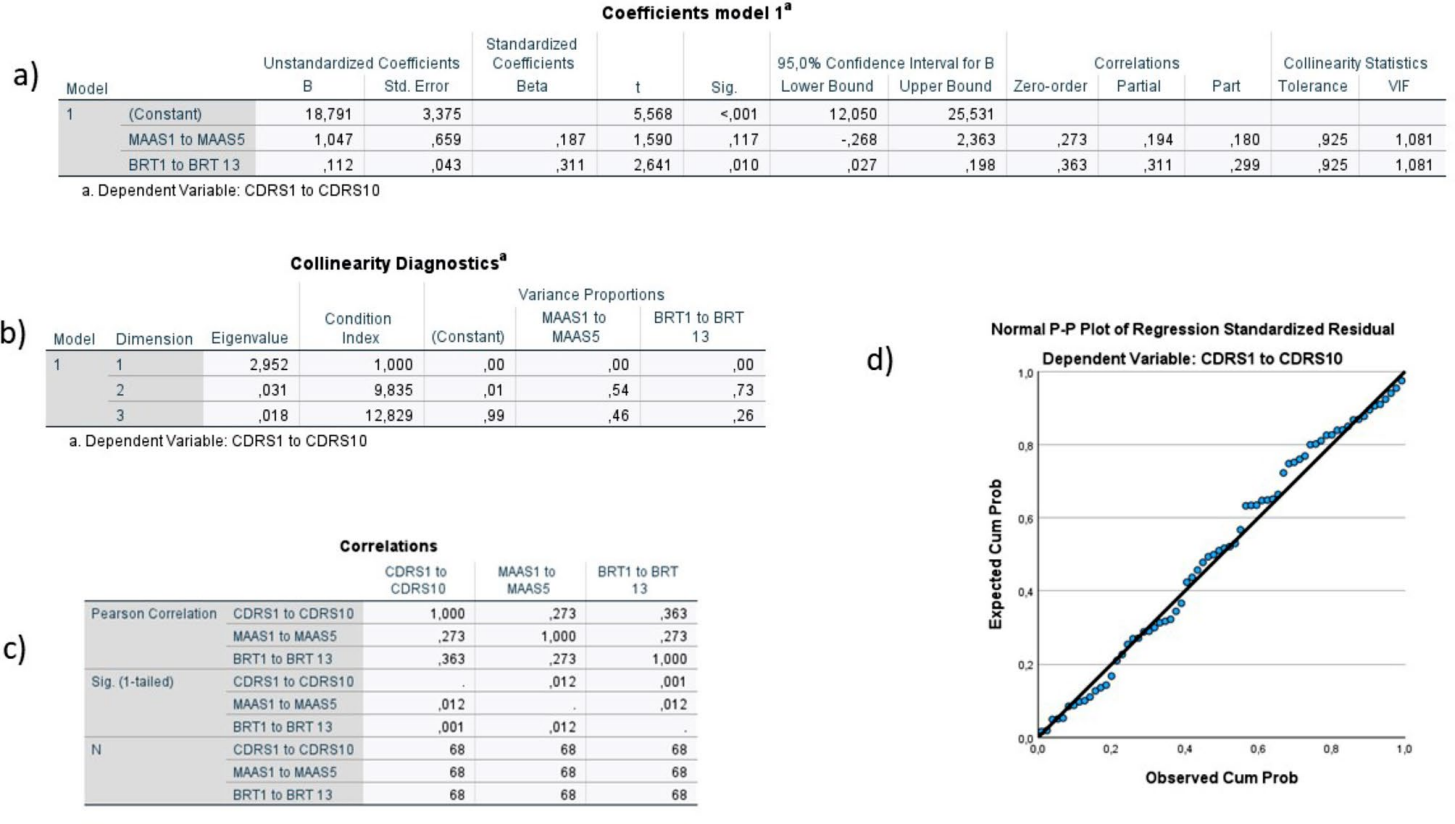
Summary of model 1. There do not appear to be any problems with multicollinearity in this model (tolerance <0.10, VIF-values >10 in the table labeled a in the figure, only one dimension with a variance proportion <0.90 in the table labeled b in the figure, and small correlation between the independent variables, the Pearson Correlation being 0.27, as seen in the table labeled c in the figure). There do not seem to be any outliers in the model, and the reasonably straight line in the Normal P-P Plot (labeled d in the figure) indicates normality of the data. The Adjusted R Square of the model is 0.138 (13.8% of the variance in individual resilience can be explained by the independent variables), which is statistically significant (F=6.38, p= 0.003). Organizational resilience contributed the largest, and statistically significant, unique contribution to the equation (Beta=0.31, p=0.01, as seen in the table labeled a in the figure)

**Fig. 10:**
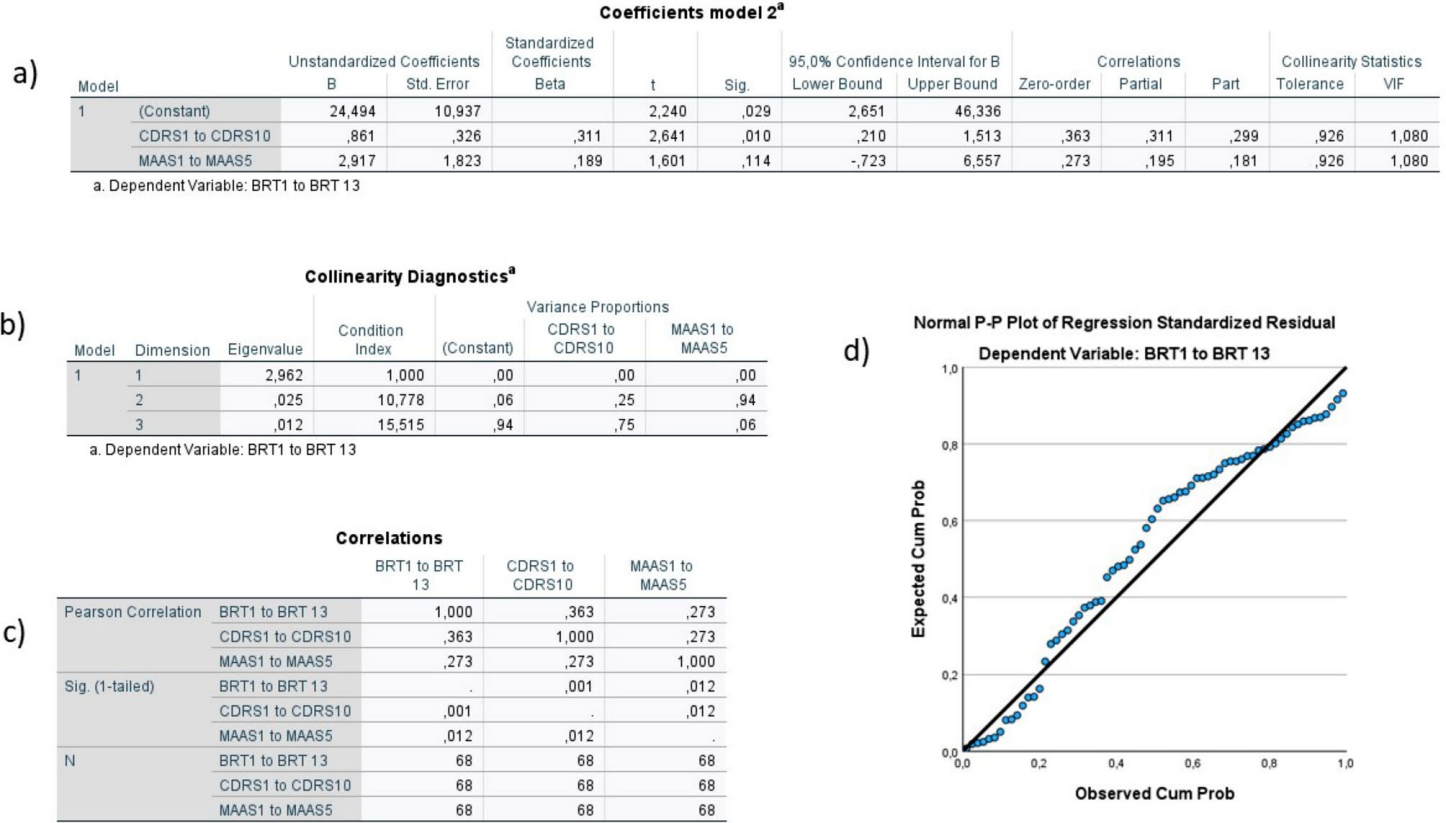
Summary of model 2There could be a small challenge with multicollinearity in this model. Tolerance <0.10, and VIF-values >10 in the table labeled a in the figure, does not indicate any problems, but there are two dimensions with a variance proportion <0.90 in the table labeled b in the figure, which can indicate some problems with multicollinearity. However, the correlation between the independent variables is low enough (Pearson Correlation =0.27) that it is not worrisome. There do not seem to be any outliers in the model, and the reasonably straight line in the Normal P-P Plot (labeled d in the figure) indicates normality of the data. The models Adjusted R Square is 0.139 (13.9% of the variance in organizational resilience can be explained by the independent variables), which is statistically significant (F=6.39, p= 0.003). Individual resilience contributed the largest, and statistically significant, unique contribution to the equation (Standardized β=0.31, p=0.01, as seen in the table labeled a in the figure)

**Fig. 11:**
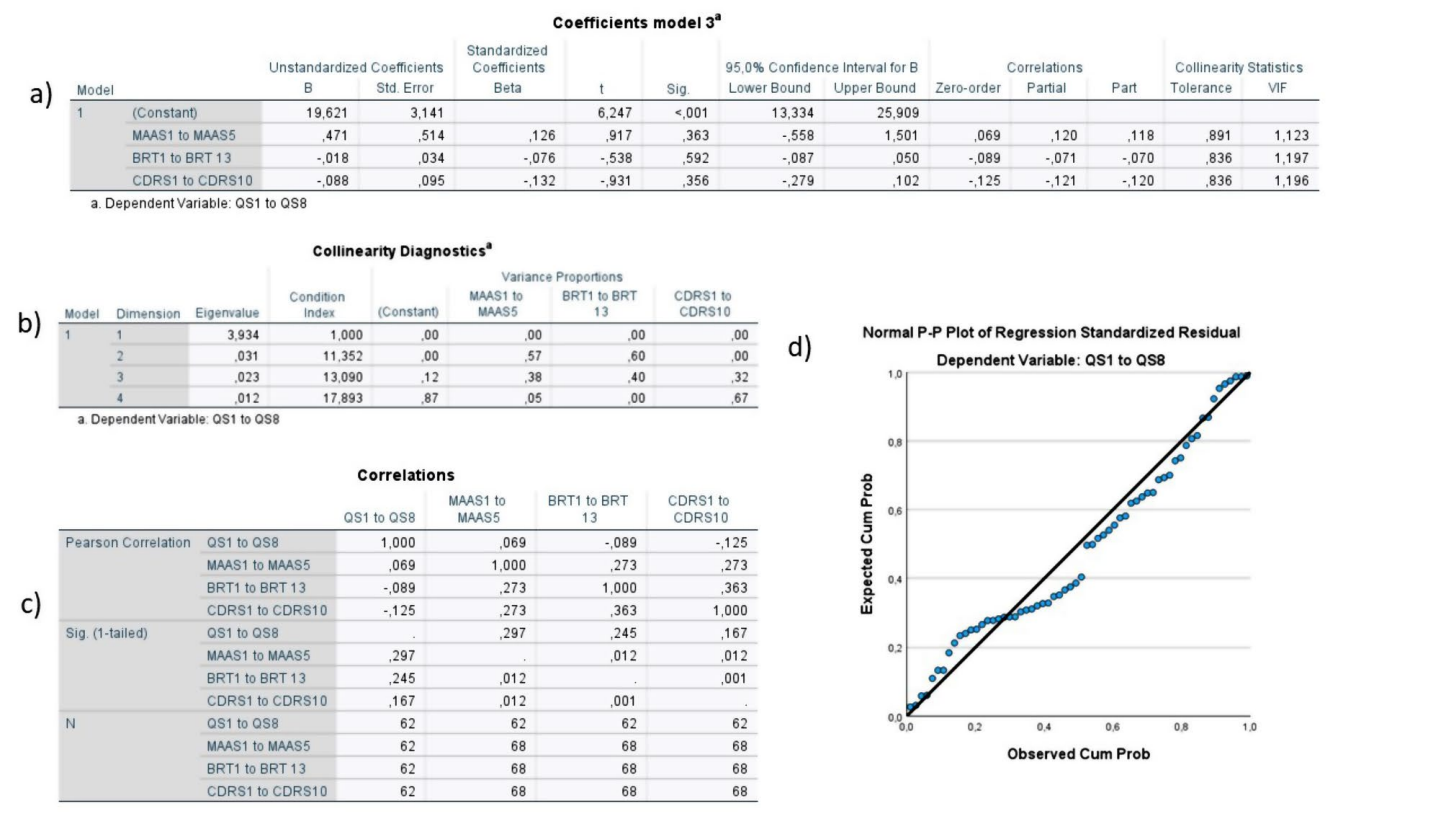
Summary of model 3There do not appear to be any problems with multicollinearity in this model (tolerance <0.10, VIF-values >10 in the table labeled a in the figure, no dimension with a variance proportion <0.90 in the table labeled b in the figure, and small correlation between the independent variables, the Pearson Correlation ranging from -0.12 to 0.07, as seen in the table labeled c in the figure). There do not seem to be any outliers in the model, and the reasonably straight line in the Normal P-P Plot (labeled d in the figure) indicates normality of the data. The models Adjusted R Square is -0.018 indicating that the independent variables do not have enough predictive value. The model is not statistically significant (F=0.64, p= 0.59).

**Fig. 12:**
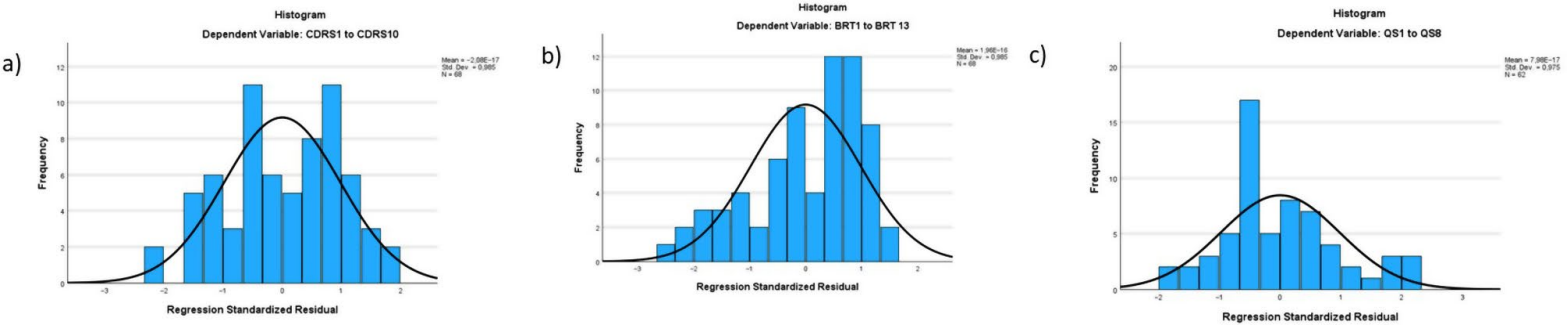
Residual analysis for model fit. Based on the residual analysis all three models have a reasonably good fit. All residuals are somewhere between -3 and 3 in all models (model 1 is labeled a in the figure, model 2 is labeled b, and model 3 is labeled c in the figure), indicating a reasonably good fit. In model 3 (labeled c), all residuals are somewhere between -2 and 2, indicating that this model might have the best fit out of the three. The residuals are also reasonably normally distributed for models 1 and 3 (labeled a and c), further supporting that the models have a good fit. For model 2 (labeled b in the figure) the residuals seem to be somewhat skewed to the left; however, they are not skewed enough that they indicate a problem with the fit of the model.

Standard multiple regression was performed to further explore the relationship between these variables, as described in the statistical analysis. The models revealed no strong violations of normality, linearity, or multicollinearity (Figs. 9, 10 and 11), and residual analysis showed model fit (Fig. 12). Model 1 showed that 13.8% of the variance in individual resilience could be explained by organizational resilience and mindfulness (adjusted R squared 0.138, intercept = 18.79, F = 6.37, *p* = 0.003, VIF = 1.08), with organizational resilience providing the largest unique contribution (β = 0.31, *p* = 0.01) (see Fig. 9 for more information).

Similar results were seen for model 2, where 13.9% of the variance in organizational resilience could be explained by individual resilience and mindfulness (adjusted R squared 0.139, intercept = 24.49, F = 6.39, *p* = 0.003, VIF = 1.08). Individual resilience provided the largest unique contribution (β = 0.31, *p* = 0.01) (see Fig. 10) Model 3 showed no statistically significant findings (adjusted R squared 0.03, intercept = 19.62, F = 0.63, *p* = 0.59, VIF = 1.12) (see Fig. 11).

## Discussion

Only a minority of respondents (12.9%) considered burnout a significant challenge among their employees, and a majority (81%) reported having a tool in place for detecting burnout. This contradicts a previous study indicating that most leaders in the radiological field consider burnout a significant challenge among their employees, with only a minority having tools available to detect burnout [21]. This difference in results could be explained by differences in what is considered a tool for detecting burnout. In this questionnaire, the respondents consider development interviews, questionnaires, and work environment surveys as tools for detecting burnout, whereas respondents in previous studies might utilize these tools, but not consider them tools for detecting burnout.

The total CD-RISC-10 and BRT 13 scores indicate relatively low individual and organizational resilience among the respondents, which is consistent with previous studies [22, 23]. This has been attributed to stress, frustration, lack of stress buffers, increased complexity of tasks, less resources, time constraints and worrying about the effect of diagnostic error on patient care [22, 23]. At the same time, these studies demonstrated a high degree of optimism, indicating confidence in respondents’ ability to overcome the difficulties at hand [22, 23].

Based on the correlation analysis there is a small, but positive relationship between the two types of resilience. This relationship is further validated through the standard multiple regression. The similar effects of individual and organizational resilience contradict a previous study showing that organizational resilience enables the resilient behavior of employees, and the capability to cope and learn at the individual level [24].

The correlation analysis further supports the claim that these are closely linked, and that it is important to take both in consideration when applying interventions to improve occupational health among healthcare workers. The need for not only individual, but also systematic, change has been demonstrated in previous studies [3–5, 8].

Although this study shows indications of relatively high mindfulness, the results regarding quality and safety demonstrate that small mistakes that can be made under stress and time constraints are still somewhat frequent. This contradicts previous studies indicating that higher mindfulness and resilience increase the quality and safety of care [3, 13]. The discrepancy may be attributable to variations in how different studies measure the quality of care. It is also possible that different studies measured mindfulness with different tools.

In conclusion: both individual and organizational resilience are somewhat low in Norwegian radiological departments, and mindfulness is somewhat high. There is a positive relationship between both types of resilience and mindfulness; however, resilience affects each other more than mindfulness. Quality and safety do not seem to be affected by either resilience or mindfulness.

### Limitations

Variables such as gender, age, and seniority (which were not included in this study) could have an effect that is not demonstrated in this study and could account for some of the differences between this and previous studies.

Another limitation of this study is the small sample size, which did not reach the suggested number of participants needed. Small sample sizes can have a negative effect on linear regression analysis, mainly affecting the validity of the results, and to some extent, the transferability of the results to other contexts.

However, both the correlation and the linear regression showed the same relationship between individual and organizational resilience, indicating that the results regrading that correlation are valid. The findings are also still transferable for quality improvement projects and future research.

Last, there were some indications of multicollinearity in model 2 (dependent variable = organizational resilience), and mindfulness might be a confounding factor with individual resilience. However, there were no strong indications for this, so the analysis was performed as planned. Due to the indications of multicollinearity and confounding being very weak, any effects of this were also expected to be minimal.

### Electronic supplementary material

Below is the link to the electronic supplementary material.

Supplementary Material 1

Supplementary Material 2

## Data Availability

The datasets used and/or analyzed during the current study are available from the corresponding author upon reasonable request.

## Abbreviations

BRT: Benchmark Resilience Tool
CDRS: Connor-Davidson Resilience Scale
MAAS: Mindfulness Attention Awareness Scale
NSD: Norwegian Social Science Data Services
SPSS: Statistical Package for Social Sciences

## References

[1] Ayyala RS, Ahmed FS, Ruzal-Shapiro C, Taylor GA (2019). Prevalence of burnout among pediatric radiologists. J Am Coll Radiol.

[2] Bundy JJ, Hage AN, Srinivasa RN, Gemmete JJ, Lee E, Gross JS (2020). Burnout among interventional radiologists. J Vasc Interv Radiol.

[3] Spieler B, Baum N (2022). Burnout: a mindful framework for the radiologist. Curr Probl Diagn Radiol.

[4] Kalantarova S, Mickinac N, Santhosh S, Malik S, Surovitsky M, Madsen L (2021). Preventing physician burnout in breast imaging: scope of the Problem and Keys to Success. Curr Probl Diagn Radiol.

[5] Ganeshan D, Wei W, Yang W (2019). Burnout in chairs of academic radiology departments in the United States. Acad Radiol.

[6] Ferguson C, Low G, Shiau G (2020). Burnout in Canadian radiology residency: a national assessment of prevalence and underlying contributory factors. Can Assoc Radiol J.

[7] Huang HL, Chen RC, Teo I, Chaudhry I, Heng AL, Zhuang KD (2021). A survey of anxiety and burnout in the radiology workforce of a tertiary hospital during the COVID-19 pandemic. J Med Imaging Radiat Oncol.

[8] Harolds JA, Parikh JR, Bluth EI, Dutton SC, Recht MP (2016). Burnout of radiologists: frequency, risk factors, and remedies: a report of the ACR Commission on Human resources. J Am Coll Radiol.

[9] Giess CS, Ip IK, Cochon LR, Gupte A, Dudley JC, Boland GW (2020). Predictors of self-reported burnout among radiology faculty at a large academic medical center. J Am Coll Radiol.

[10] Giess CS, Ip IK, Gupte A, Dudley JC, Healey MJ, Boland GW (2022). Self-reported burnout: comparison of radiologists to nonradiologist peers at a large academic medical center. Acad Radiol.

[11] Shields M, James D, McCormack L, Warren-Forward H (2021). Burnout in the disciplines of medical radiation science: a systematic review. J Med Imaging Radiation Sci.

[12] Fennessy FM, Mandell JC, Boland GW, Seltzer SE, Giess CS (2021). Strategies to increase resilience, team building, and productivity among radiologists during the COVID-19 era. J Am Coll Radiol.

[13] Melo JACd, Gelbcke FL, Amadigi FR, Huhn A, Silva Cd, Ribeiro G. Psychological exhaustion of radiological nursing workers in nuclear medicine services. Revista Brasileira De Enfermagem. 2021;73. 10.1590/0034-7167-2020-0169 33470380

[14] Serrat O. On resilient organizations. 2013.

[15] Liang F, Cao L (2021). Linking employee resilience with Organizational Resilience: the roles of coping mechanism and managerial resilience. Psychol Res Behav Manage.

[16] Southwick FS, Martini BL, Charney DS, Southwick SM, Marques J, Dhiman S (2017). Leadership and Resilience. Leadership Today: practices for Personal and Professional Performance.

[17] Patriarca R, Di Gravio G, Costantino F, Falegnami A, Bilotta F (2018). An Analytic Framework to assess Organizational Resilience. Saf Health Work.

[18] Campbell-Sills L, Stein MB (2007). Psychometric analysis and refinement of the connor–davidson resilience scale (CD‐RISC): validation of a 10‐item measure of resilience. J Trauma Stress: Official Publication Int Soc Trauma Stress Stud.

[19] Brown KW, Ryan RM (2003). The benefits of being present: mindfulness and its role in psychological well-being. J Personal Soc Psychol.

[20] Whitman R, Kachali Z, Roger H, Vargo D, Seville J (2013). Short-form version of the Benchmark Resilience Tool (BRT-53). Measuring Bus Excellence.

[21] Parikh JR, Bender CE (2021). How Radiology leaders can address Burnout. J Am Coll Radiol.

[22] Huang L, Wang Y, Liu J, Ye P, Cheng B, Xu H (2020). Factors associated with resilience among medical staff in radiology departments during the outbreak of 2019 novel coronavirus disease (COVID-19): a cross-sectional study. Med Sci Monitor: Int Med J Experimental Clin Res.

[23] Sood A, Sharma V, Schroeder DR, Gorman B (2014). Stress management and resiliency training (SMART) program among Department of Radiology faculty: a pilot randomized clinical trial. Explore.

[24] Gröschke D, Hofmann E, Müller ND, Wolf J. Individual and organizational resilience—insights from healthcare providers in Germany during the COVID-19 pandemic. Front Psychol. 2022;13. 10.3389/fpsyg.2022.965380 PMC9453859 36092080

