# Supplementary material for: Resilience and mindfulness among radiological personnel in Norway, their relationship and their impact on quality and safety– a questionnaire study
Source: BMC Res Notes. 2024 Apr 1;17:96. doi: 10.1186/s13104-024-06748-1 (PMC10983646; doi:10.1186/s13104-024-06748-1)
Supplement: Supplementary file 1 — Supplementary Material 1 [file 13104_2024_6748_MOESM1_ESM.pdf]

Interview guide for pilot study for questionnaire validation

Information given at the start of the conversation: The survey you have filled in will be anonymous, and will be deleted after the interview. The information used will be your views on the questions and any opportunities for improvement.

Review of the consent form, focusing on the fact that they will remain anonymous and that they can withdraw their consent at any time.

Questions to be used during the interview:

- How did you experience the questions in the questionnaire?

- Were there any words you wondered what they meant or what the meaning behind them was?

- Were there any words or terms that it is not natural for you to use, which you recommend that we replace?

- Did you possibly have any thoughts on what these terms could be replaced with that would be more natural for you to use?

- Were any of the questions unclear or clear? (here I mean that you were unsure what to answer)

- Did you feel that the questions were relevant to how your workplace operates? (possible auxiliary question - which were most relevant, and which were least relevant?

- Did you feel that some of the questions were inappropriate, or that you could no longer be anonymous if you answered them (help questions: could it be easy to find back to you and where you work by looking at how you have answered some questions?

- How long did it take you to answer the survey?

- Is there anything else you would like to point out that we can do to get this questionnaire as good as possible?
