# Supplementary material for: Resilience and mindfulness among radiological personnel in Norway, their relationship and their impact on quality and safety– a questionnaire study
Source: BMC Res Notes. 2024 Apr 1;17:96. doi: 10.1186/s13104-024-06748-1 (PMC10983646; doi:10.1186/s13104-024-06748-1)
Supplement: Supplementary file 2 — Supplementary Material 2 [file 13104_2024_6748_MOESM2_ESM.pdf]

Questionnaire – Resilience, mindfulness, quality and safety

Thank you for taking the time and agreeing to take part in this survey! No information that can identify you will be collected in this survey. We would also like to remind you that you can withdraw from this study at any time.

This survey has been developed by AMG as part of a research project at SHARE - Center for quality and safety in the health service (University of Stavanger). The aim of this survey is to get an overview of how you as an individual handle stress and crises, how the organization you work in handles unexpected events and crises.

Part 1 consists of some questions where the purpose is to collect some background information about your workplace (private/public and size). It is also a question of what type of position you have and whether you have personnel responsibility or not.

Part 2 consists of some questions about how you yourself handle changes, unexpected events, stress and difficult situations. In this research, this is called resilience.

Part 3 consists of some questions about your own awareness of what is happening in the moment, and how well you manage to focus on what is happening in the moment. In this survey, this is called mindfulness.

Part 4 is about how the organization you work in handles unexpected events and crises. It is also a bit about how you handle going from a crisis back to normal operations.

There are also some questions about quality and safety that are only for radiographers, and some questions that are only for those with personnel responsibilities about how employee burnout is detected and handled.

Overall, this survey will take about 30 minutes to answer. When you are ready, you can press next to get to part 1 of the survey.

Part 1 – Background variables

In this section, we would like to get some background information about your workplace. An example of this is if you work at a public hospital or a private organisation. You will also be asked about the type of position you have and which stress management exercises you used, if you used any of them.

1. Do you work in a department at a public hospital, in a private institution, or both?
2. How big is your department in terms of number of labs? (This also includes CT, MRI, and intervention)
3. What position do you hold? (radiographer, radiation therapist, radiologist or trainee)
4. Does your position include personnel management?

Part 2 – Individual Resilience

In this section, we want to find out something about how you yourself handle changes, unexpected events, stress and difficult situations, i.e. your individual resilience. In this context, resilience means the ability to prepare for and plan for, bounce back from and be better equipped to adapt to unwanted events.

This part consists of 10 questions, and is about how you handle unexpected events and changes, stress and unpleasant feelings. All questions are answered on a scale from 0-4 where 0 = not true at all, 2 = somewhat true, and 4 = almost always true.

We expect you to spend about five minutes answering this part.

1. I am able to adapt when changes occur.
2. I can deal with whatever comes my way.
3. I try to see the humorous side of things when I am faced with problems.
4. Having to cope with stress can make me stronger.
5. I tend to bounce back after illness, injury or other hardships.
6. I believe I can achieve my goals, even if there are obstacles.
7. Under pressure, I stay focused and think clearly.
8. I am not easily discouraged by failure.
9. I think of myself as a strong person when dealing with life's challenges and difficulties.
10. I am able to handle unpleasant or painful feelings like sadness, fear, and anger.

Part 3 - Awareness of what is happening in the moment

In this section, we want to know something about how well you are able to focus on what is happening in the moment, how attentive you are to the tasks you are doing, and whether you experience doing things on "autopilot". In this connection, we call this mindfulness.

This part consists of 5 questions, all of which must be answered on a scale from 1 - 6, where 1 = almost always and 6 = almost never. When you answer these questions, take your previous working day as a starting point, and state which answer fits best with what you experienced.

1. I was finding it difficult to stay focused on what was happening.
2. I was doing something without paying attention.
3. I was preoccupied with the future or the past.
4. I was doing something automatically, without being aware of what I was doing.
5. I was rushing through something without being really attentive to it.

Part 4 - Organizational resilience

In this section, we want to know something about how the organization you work in handles crises and unexpected events, the network you are part of, and how you adapt between crisis management and normal operations.

This part consists of 13 questions, all of which must be answered on a scale from 1 - 8, where 1 = completely disagree, and 8 = completely agree. We expect it to take about 5 minutes to answer this part.

1. We are familiar with how a crisis can affect us
2. We believe that contingency plans need to be practiced and tested in order to be effective
3. We are able to adapt quickly from normal operations to responding to crises
4. We develop relationships with organisations that we may have to work with during a crisis
5. Our priorities for restoring normal operations will provide our employees with a direction during a crisis
6. There is a sense of teamwork and camaraderie in our organisation
7. Our organisation has adequate resources for being able to handle unexpected changes
8. The people in our organisation "own" a problem until it has been resolved
9. Our employees have the information and knowledge they need in order to respond to unexpected problems
10. Our organisation's managers take the lead by leading by example
11. Our employees are rewarded for 'thinking outside the box'
12. Our organisation is able to make tough decisions quickly
13. Our managers actively listen for problems

How is employee burnout detected, and is it something that is addressed

In this section, we want you to say something about your experience of the employees' well-being at work, which stress factors affect the employees, whether there are any tools used to detect burnout, and whether there is anything that is taken up.

This part consists of 5 questions, and takes about 5 minutes to answer.

1. To what degree is burnout a problem among you employees?
2. In your opinion, to what extent do personal/social stressors affect your employees' well-being?
3. Do you use any tools to assess employee well-being and detect burnout?
4. If yes, which tool do you use?
5. How do you rate your own effectiveness in regards to detecting burnout among your employees?

Quality and safety

In this part of the survey, I want to find out something about how you experience the quality of the work done. The things I am asking about are things that can be assumed to be affected by stress, such as wrong positioning, wrong labeling of images, contrast security etc.

All questions are answered on a scale from 1 - 5, where 1 = Never, and 5 = very often. This section consists of eight questions, and we expect it to take about five minutes to answer.

1. How often do you think retakes of images are done in your department due to positioning errors?
2. How often do you think retakes of images are done in your department due to the wrong side being imaged (right ankle instead of the left ankle for example)?
3. How often do you think retakes of images are done in your department due to the wrong protocol / procedure being used?
4. How often do you think retakes of images are done in your department due to incorrect collimation?
5. How often do you think images are marked wrong in your department? For example that the image is marked as right side instead of left, lacking marking for whether the image was taken with the patient standing, sitting, lying down, trauma, etc.
6. How often do you think “double imaging” is done in your department? This can for example be due to lack of information about previous imaging, or lack of access to previous images.
7. How often do you think intravenous contrast ends up going subcutaneously in your department?
8. Does it ever happen that confirming the patient’s identity through asking about data of birth is forgotten about in your department?

Finishing statement

Are there any other factors surrounding any of the themes this questionnaire has asked you about (resilience, quality and safety) that has not come up that you feel it is important that I know about?
